# New reporters for monitoring cellular NMD

**DOI:** 10.1261/rna.080272.124

**Published:** 2025-04

**Authors:** Hanna Alalam, Monika Šafhauzer, Per Sunnerhagen

**Affiliations:** Department of Chemistry and Molecular Biology, University of Gothenburg, 413 90 Göteborg, Sweden

**Keywords:** *Saccharomyces cerevisiae*, genetic screening, nonsense-mediated decay, ribosome recycling

## Abstract

Nonsense-mediated decay (NMD) is a eukaryotic surveillance pathway that controls degradation of cytoplasmic transcripts with aberrant features. NMD-controlled RNA degradation acts to regulate a large fraction of the mRNA population. It has been implicated in cellular responses to infections and environmental stress, as well as in deregulation of tumor-promoting genes. NMD is executed by a set of three core factors conserved in evolution, UPF1-3, as well as additional influencing proteins such as kinases. Monitoring NMD activity is challenging due to the difficulties in quantitating RNA decay rates in vivo, and consequently, it has also been problematic to identify new factors influencing NMD. Here, we developed a genetic selection system in yeast to capture new components affecting NMD status. The reporter constructs link NMD target sequences with nutrient-selectable genetic markers. By crossing these reporters into a genome-wide library of deletion mutants and quantitating colony growth on a selective medium, we robustly detect previously known NMD components in a high-throughput fashion. In addition, we identify novel mutations influencing NMD status and implicate ribosome recycling as important for NMD. By using our constructed combinations of promoters, NMD target sequences, and selectable markers, the system can also efficiently detect mutations with a minor effect, or in special environments. Furthermore, it can be used to explore how NMD acts on targets of different structures.

## INTRODUCTION

Nonsense-mediated decay (NMD) was originally described as a mechanism in eukaryotes for the recognition and degradation of mRNAs with premature termination codons (PTCs; “nonsense codons”) in the interest of preventing their translation into truncated and potentially toxic proteins (Losson and Lacroute 1979). Other mRNA structures can also trigger NMD, e.g., long 3′ untranslated regions (UTRs) or splice junctions downstream from the main open reading frame (ORF) (Goetz and Wilkinson 2017). A large fraction, estimated at 10%–20%, of all mRNAs can be regulated directly or indirectly through NMD (Guan et al. 2006; Celik et al. 2017).

A core set of proteins required for NMD have been identified. Upf1 is the central partner, an mRNA-binding RNA helicase conserved in evolution and generally viewed as the executing component (Lavysh and Neu-Yilik 2020). Upf1 physically interacts with two other conserved proteins, Upf2 and Upf3, leading to its activation. Phosphorylation of Upf1 at multiple sites strongly enhances its activity. In mammals, this phosphorylation is carried out by the serine/threonine protein kinase SMG1. In yeast, Upf1 is also phosphorylated, but the kinase has not been identified and a strict requirement for phosphorylation has not been established. Beyond this conserved core set, additional NMD factors have been found that vary between organisms (Kurosaki et al. 2019).

In mammalian and plant cells, the NMD pathway functions as a modulator of pathways responsive to infection and cellular stress (Goetz and Wilkinson 2017). Many viral RNA genomes and transcripts contain features that are nonstandard in their eukaryotic hosts, and which can become targets for NMD. There is reason to believe that cellular NMD is a major host defense factor against viral infection in eukaryotes, including mammals (Balistreri et al. 2014) and vascular plants (Garcia et al. 2014). In cancer cells, NMD-triggering mutations such as PTCs are enriched (Lindeboom et al. 2016). The set of proteins encoded by NMD-targeted transcripts is enriched for stress responses and tumor-promoting pathways (Wang et al. 2011). *Arabidopsis* transcripts down-regulated by NMD are primarily implicated in responses to pathogens and environmental stress (Rayson et al. 2012). These considerations show that it can be relevant to identify new factors impinging on NMD in order to combat human diseases such as viral infections or cancer, as well as improving stress tolerance in plants and microorganisms.

Identifying proteins affecting NMD is difficult with existing techniques. It has not been possible to reconstitute a functional NMD in vitro system, leaving genome-wide mutational analysis as the only viable approach for unbiased searches. If an NMD component is mutated, this can be detected through a half-life change of its target RNAs. However, the search for NMD components outside of the core set has been hampered by the lack of fast and reliable methods to quantitate decay rates for individual RNA species. Consequently, the change in the steady-state level of a transcript is often used as a proxy for the change of its half-life, if it can be assumed that the transcription rate does not change. Determination of steady-state levels of individual mRNAs by qPCR is a precise but labor-intensive method. Recently, accurate methods for decay rates involving metabolic RNA labeling have been developed (Russo et al. 2017; Alalam et al. 2022). Those methods are well suited for global analyses, but would be cumbersome to apply for individual RNA species.

Thus, identifying novel NMD components through a genome-wide screen for mutants defective in NMD requires a way to transform that defect into a directly selectable phenotype. Further, a positive selection mode is preferable for screening, since the numerous mutations with a nonspecific negative impact on growth can be filtered out. Here, we have designed a series of genetic selection constructs in *S. cerevisiae* intended to identify proteins able to influence the cellular NMD status. Each construct consists of a specific combination of a promoter, driving expression of a selectable marker gene, flanked by known NMD target sequences. A large number of constructs were designed and tested in order to identify optimal combinations for detection of putative NMD factors with large or small phenotypic effects, as well as for different expression levels. For a genome-wide screen, the selection construct is then combined with a library of gene deletions, and the entire set of mutants is cultured under selective conditions, where expression of the reporter gene is limiting for growth. Construction of genome-scale libraries is simplified using a high-efficiency crossing scheme (Reid et al. 2011) to achieve maximal throughput, and the growth of individual mutants is monitored as colonies on agar in an automated, highly parallel system (Zackrisson et al. 2016).

In this work, as proof of principle we demonstrate that our genetic selection system, in a genome-wide screen, can detect already known NMD components with major or minor influence on NMD activity. As a new NMD-influencing factor, we identify mutants lacking the Rab protein Vps21, required for vesicle transport to the vacuole, as defective in degrading the well-characterized NMD target unspliced *RPL28* mRNA (Swida et al. 1986). Functional vacuoles are required for iron uptake and by extension synthesis of the ribosome recycling factor Rli1, a Fe–S cluster-containing protein. We also detect the ribosome recycling factor mutation *tma20*, which was independently reported recently (Pacheco et al. 2024). These two findings implicate ribosome recycling as an essential component of NMD. Our selection systems can also be used to search for components with a weak effect or conditionally required for NMD. To detect components required only under certain conditions and to detect environmental effects on NMD efficiency, screening can be done by applying various external stresses. It is further possible to screen for small molecule NMD inhibitors, and for the action of NMD on targets of different structures. In summary, we have established a flexible and high-throughput genetic platform in yeast to monitor cellular NMD status.

## RESULTS

### Design and initial testing of the system

We aimed to design a simple system to monitor the NMD status of cells. It was previously demonstrated that mutations of the carbamoyl phosphate synthetase (CPSase) domain of the Ura2 protein cause a slow growth upon the addition of exogenous arginine, due to reduction of the intracellular pool of carbamoyl phosphate (Messenguy et al. 2002). Usually, carbamoyl phosphate is produced by the two enzymes Ura2 and Cpa1. The *URA2* gene expression and Ura2 protein activity are regulated by negative feedback loops controlled by the level of pyrimidines, while expression of the *CPA1* gene is repressed in response to the level of arginine. A mutation compromising the ability of Ura2 to produce carbamoyl phosphate would lead to a reduction in growth rate in media containing arginine but lacking pyrimidines due to the reduction of the carbamoyl phosphate pool due to the concurrent repression of *CPA1* by arginine. However, the arginine-based repression of *CPA1* is effected through NMD, hence an NMD-defective mutant is able to grow more robustly in arginine-containing media due to a higher level of Cpa1 (Messenguy et al. 2002). This system can be visualized in Supplemental Figure S1.

We wanted to test whether a system based on NMD-mediated *CPA1* inhibition would provide reasonable sensitivity to detect mild changes in NMD. Hence, a *URA2* allele lacking the majority of the protein with the exception of the linker region after the dihydroorotase (DHOase)-like domain and the aspartate transcarbamoylase (ACTase) domain (Souciet et al. 1989) was constructed in plasmid pHLUMv2 (Addgene #64166), which complements all the auxotrophies of the BY4741 strain. The resulting plasmid (pHA_URA2ΔCPSaseV3) was transformed into the BY4741 strain and the isogenic *upf3*Δ strain. In both cases, the wild type (wt) *URA2* locus was deleted. Both strains were grown in minimal media lacking all amino acids and supplemented either with ammonium sulfate or arginine as sole nitrogen sources. We applied two criteria to determine the reporter's suitability for genome-wide NMD screening. First, we asked whether the observed difference was biologically relevant for screening the NMD pathway, and second, whether the change in growth rate was sufficient for a comprehensive genomic screen at a reasonable sensitivity. These considerations led to the following requirements: for the reporter to be deemed significant, it must exhibit a growth-rate change >15% in a strain carrying a core NMD-factor mutation. Moreover, for the reporter to be deemed practical, it must exhibit a growth-rate change greater than 50% in that same mutated strain, when grown with arginine as a sole nitrogen source. Based on the criteria above, the *upf3*Δ *ura2*ΔCPSaseV3 strain showed significantly better growth relative to *UPF3 ura2*ΔCPSaseV3 (Supplemental Fig. S2) under the condition where arginine was the sole nitrogen source (26.5% increased growth in a *upf3*Δ strain relative to wt). However, the modest separation between the strains was insufficient to be practical for a genome-wide screen, especially considering that the *upf3*Δ mutation fully inactivates the NMD pathway. This raises concerns about the sensitivity of the system to detect subtle changes to NMD. Therefore, we decided to investigate an alternative system.

We reasoned that the use of transcripts known to be regulated by NMD through a non-PTC feature would serve as a convenient starting point to construct a sensitive NMD detection system. Initially, we envisioned that the system would be based on the differential toxicity of the bacterial endoribonuclease toxin MazF, which we successfully utilized for other screening purposes (Alalam et al. 2021). In this configuration, mutations or conditions that cause a reduction in NMD activity causes slower growth due to the increased stability of the *mazF* transcript. However, we were unable to transform the plasmid in either wt or *upf3*Δ mutant even when using the weak *ALR1* promoter, indicating that the toxicity was too high to serve any practical screening purpose. The design of the toxin-based systems can be found in Supplemental Figure S3.

**FIGURE 1.**
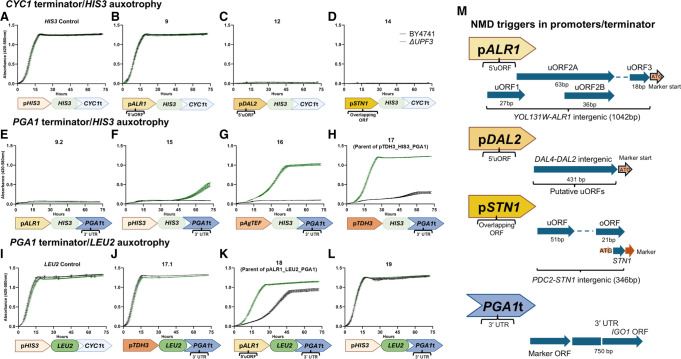
NMD monitoring based on auxotrophic markers. Various plasmids carrying combinations of promoters, markers, and terminators were transformed into BY4741 and isogenic *upf3*Δ. Growth measurements are presented as mean ± standard error of the mean for each time point. Optical density values were produced by Bioscreen for each strain in biological triplicates. The number on *top* of each graph designates the specific combination of regulative elements used to construct a given plasmid. These are shown schematically at the *bottom* of each panel, and in more detail in panel *M*. Plasmids that were used to derive the plasmids for screening are shown with the name of the derived plasmid in parentheses.

The negative-selection screening scheme thus proved problematic. Therefore, we instead considered a positive selection screening scheme that leverages the preexisting auxotrophies present in the commonly used parental strain of the deletion library (BY4741). In this configuration, conditions or mutations that increase the half-life of an NMD-sensitized transcript encoding the auxotrophy gene allows for enhanced growth relative to the wt (or basal condition). We started our testing by using the *HIS3* marker as the auxotrophy reporter, since 3-amino-1,2,4-triazole (3-AT) can be used to accentuate the growth difference if needed. Initially, three promoters were selected to drive the *HIS3* marker: the *ALR1* promoter triggers NMD through 5′ uORFs (Johansson and Jacobson 2010), the *DAL2* promoter contains a putative upstream 5′ uORF that is likely to act as an NMD trigger (He et al. 2003), and the *STN1* promoter contains an overlapping ORF that triggers NMD (Torrance and Lydall 2018). The wt *HIS3* promoter is not known to trigger NMD and was used as a positive control. All constructs were terminated by the *CYC1* terminator, which is not an NMD trigger (Fig. 1; Alalam et al. 2022).

Plasmids were constructed in pCM188 backbone (*URA3* selection) based on the reporters above and transformed in both wt and *upf3*Δ backgrounds, and their growth assessed in -URA -HIS media using Bioscreen (Warringer and Blomberg 2003), and the same criteria that were utilized for assessing plasmid pHA_URA2ΔCPSaseV3 were applied to the new constructs. The *ALR1*-based construct (NMDv9) did not show a significant difference between the strains (Fig. 1B; growth rate change of 13.6%) unless 3-AT was added (Supplemental Fig. S4), which resulted in a significant change of the growth rate by 25.8% and 94.1% for 0.5 and 1 mM 3-AT, respectively. Both the *DAL2* (NMDv12) and *STN1* (NMDv14) based constructs failed to support growth (Fig. 1C,D), indicating inadequate levels of expression of *HIS3*. This indicated that neither of these constructs was suited for the screening, and although NMDv9 met all our usage criteria, it caused a significant decline in the overall growth rate, unnecessarily prolonging the screening time. Hence, we looked for an alternative NMD-triggering feature for further testing. The *PGA1* transcript has previously been shown to be NMD-regulated due to its 750 bp long 3′ UTR, which extends into the downstream *IGO1* gene (Kebaara and Atkin 2009). This 3′ UTR was used for the construction of another set of reporters. Same as before, the *HIS3* marker was coupled to various promoters in increasing order of strength: *ALR1*, *HIS3*, *Ashbya gossypii TEF1*, and *TDH3*; and tested in -URA -HIS media. Under these conditions, the *ALR1*-based construct (NMDv9.2) failed to support growth, most likely due to low expression from the *ALR1* promoter combined with a strongly reduced half-life of the transcript stemming from having two NMD-triggering features in the same transcript (Fig. 1E). For the other constructs, we observed increased growth only in *upf3*Δ, correlating with the strength of the promoter (Fig. 1F–H). The growth was most robust with the *TDH3* expression-based reporter, whereas the wt did not exhibit any growth for the initial 48 h (NMDv17, Fig. 1H).

The second set of constructs provided a strong and clear separation between the strains; however, we were interested in creating additional constructs that allow for additional comparisons since the *TDH3* and *TEF1* constructs have a high expression level, and we wanted to compare to low levels of expression as well. To this end, we turned our attention to the selection marker and constructed plasmids that used *LEU2* as a reporter instead of *HIS3* while retaining the *PGA1* 3′ UTR. Under these conditions, we determined that unlike *HIS3*, the *TDH3*-driven *LEU2* construct (NMDv17.1) displayed similar growth between wt and *upf3*Δ (Fig. 1J), while the combination of both the *ALR1* promoter and the *PGA1* 3′ UTR (NMDv18) provided appreciable separation between the strains (86.9% change in growth rate) and allowed a level of background growth of the wt, hence allowing for a quantitative approach (Fig. 1K). Finally, increasing the expression of *LEU2* using the *HIS3* promoter while retaining the *PGA1* 3′ UTR led to a complete loss of the separation (NMDv19, Fig. 1L; growth rate change of 4.1%). These results indicate that the required amount of the Leu2 protein for cellular growth is lower than the amount of the His3 protein, and that the combination of both an NMD-sensitive 5′ UTR from *ALR1* and an NMD-sensitive 3′ UTR from *PGA1* is sufficient to create a suitable screening system when combined with the *LEU2* marker. All growth rates and their associated change percentages of the reporters in which both wt and *upf3*Δ showed appreciable growth can be found in Supplemental Table S1.

### Library construction and screening

We began the construction of the NMD reporter library by exchanging the *URA3* marker carried on the NMDv18 reporter for a *HIS3* selection (pALR1_LEU2_PGA1). This was done to make the plasmid compatible with our library construction method, which involves 5-FOA counterselection (Reid et al. 2008), hence rendering plasmids carrying the *URA3* marker unsuitable for high-throughput construction. Subsequently, the previously described selective ploidy ablation protocol (Reid et al. 2011) was utilized to transfer plasmid pALR1_LEU2_PGA1 into the *S. cerevisiae* BY4741 deletion library.

The NMD reporter library was screened using the Scan-o-matic framework (Zackrisson et al. 2016). In this setup, arrayed colonies are scanned repeatedly using flatbed scanners for the duration of the experiment, and the pixel counts at each position are converted to cell numbers, which allows for the construction of a growth curve of each mutant using the calculated cell numbers from consecutively scanned images. We compensated for inherent differences in the growth rate between mutants by conducting the screen at both basal conditions (only selection for plasmid, not for NMD reporter expression) and test condition (selection of both plasmid marker and NMD reporter expression) and normalizing for the growth rate relative to the control calculated from each condition. This correction yields a finalized interaction score, which accounts for differences in mutants’ growth rate and indicates enhanced growth when the value is positive or reduced growth when the value is negative (see Materials and Methods for details).

The distribution of interactions of yeast knockout (YKO) mutants (median of each mutant replicates) and control strain (BY4741) are shown in Supplemental Figure S5. The control had a sharper peak and narrower distribution, as expected, and the peak of both the YKO mutants and control was at approximately zero, which indicates that there was no systematic effect on the reporter in the majority of the mutants (data from primary screen can be found in Supplemental Table S2). Next, we used stringent selection criteria to select mutants showing a relative enhanced growth under the test condition. To fulfill the criteria, mutants would have a *Q* value of <0.05, an interaction score of higher than 2 × median absolute deviations from the median of the control, and a coefficient of variation of ≤0.35, OR that the lowest value for replicates of a given mutant did not fall below the interaction magnitude cutoff designated above. Seventy-three mutants meeting the criteria above were designated primary screen candidates (Supplemental Table S3), and 58 of them were rescreened in a secondary screen but with a doubled number of replicates compared to the primary screen (*n* = 12). ORFs that were eliminated from analysis for various reasons can be found in Supplemental Table S4.

**FIGURE 2.**
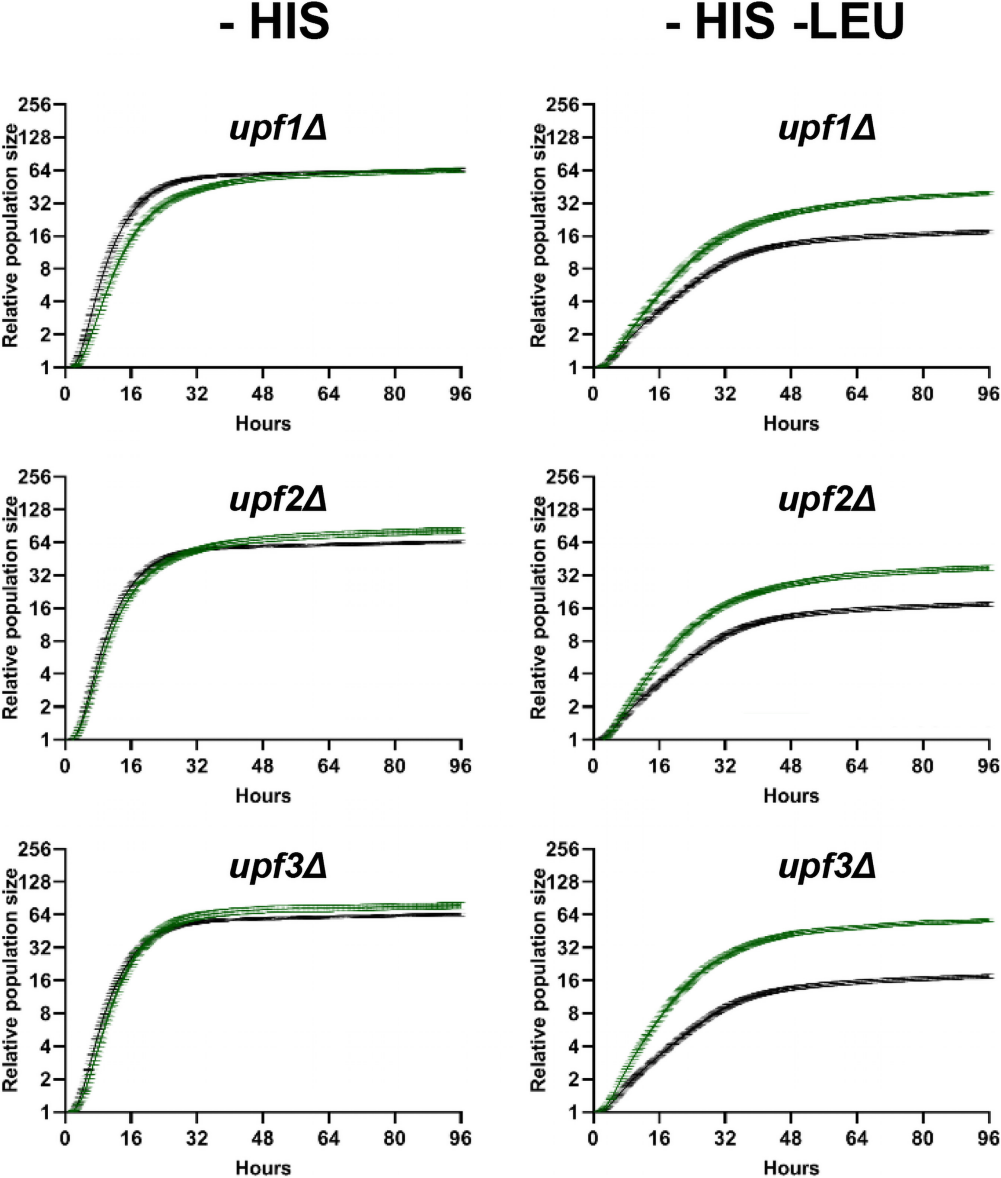
Growth curves of strains with deleted core NMD factors using pALR1_LEU2_PGA1. Growth curves of core NMD factors from the secondary screen of the first library. Green curve, mutant; black curve, wt. Each growth curve was normalized to the initial population size. Growth measurements are presented as mean ± standard error of the mean for each time point for 12 biological replicates. All the core mutants passed the criteria for significance (*Q* value of <0.05, an interaction score of higher than 2 × median absolute deviations from the median of the control, and a coefficient of variation of ≤0.35). Methodology for calculating the interaction score can be found in Materials and Methods.

The secondary screen was conducted in a similar manner to the primary screen and the same filters for candidate selection were applied. Upon secondary screening, a total of 34 mutants tested positive using the cutoffs mentioned previously (Supplemental Table S5). The growth curves from the secondary screen of core NMD-factor mutants are shown in Figure 2.

Next, we constructed an additional NMD library that expresses the NMD reporter at a higher level using plasmid pTDH3_HIS3_PGA1, which is identical to plasmid NMDv17 with the exception of exchanging the *URA3* marker for a *LEU2* marker for the same reason as described above. The second library was constructed similar to the first library with the exception of modifying the drop-out media to suit the *LEU2* marker on the plasmid. The second library was screened qualitatively using Scan-o-matic. Mutants were assayed in quadruplets and those displaying appreciable growth by 48 h upon visual inspection were considered positive. The screen of the second library was limited to 48 h since at that point background growth of the wt strain complicates the interpretation of the results. Mutants identified as positive in the primary screen of the second library can be found in Supplemental Table S6.

**FIGURE 3.**
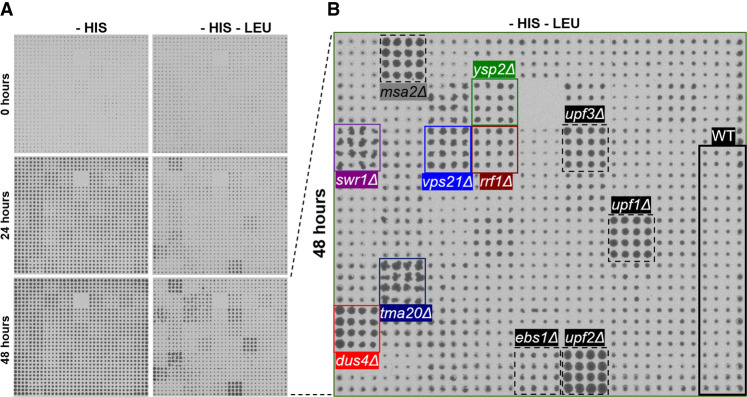
Finalized qualitative screen using pTDH3_HIS3_PGA1. (*A*) Image at the start of the experiment and at 24 and 48 h intervals are shown. (*B*) Image at the end of the experiment under the selective condition at 48 h is shown (magnification of *bottom right-hand* field in *A*. Mutants affected in previously known NMD factors are highlighted (black), as well as candidates that show strong phenotype and were used for further experiments: *ysp2*Δ (green), *swr1*Δ (purple), *vps21*Δ (blue), *rrf1*Δ (brown), *tma20*Δ (dark blue), *dus4*Δ (red). Colonies marked in stippled black represent a misplaced strain (*msa2*Δ) and were ignored.

Surprisingly, there was little overlap between the screens of the first and second library outside of the core NMD factors and one mutant (*tuf1*Δ). Moreover, factors that weakly influence NMD, namely *nmd4*Δ and *ebs1*Δ, were only detectable using the second library (pTDH3_HIS3_PGA1). Hence, we decided to carry out a finalized screen that pooled the positive hits from the secondary screen of the first library and the second library using the more sensitive pTDH3_HIS3_PGA1 plasmid to rule out candidates that are likely affecting the expression of *ALR1* rather than NMD. The finalized screen was conducted similarly to the qualitative screen used for the second library with the modification of using a higher number of replicates (*n* = 16) and assisting the visual inspection with synthetic gene array (SGA) tools (Wagih et al. 2013). The finalized screening plate is shown at time points 0, 24, and 48 h in Figure 3A. An additional growth curve for *nmd4*Δ using Bioscreen was added to rule out the subjective bias associated with calling out weak candidates visually (Supplemental Fig. S6). The growth of some candidates chosen for further experiments is shown in Figure 3B. The finalized results are provided in Supplemental Table S7, and the layout of the plate can be found in Supplemental Table S8.

### Comparison of NMD screens and selection of further candidates for confirmation

The pALR1_LEU2_PGA1 plasmid displayed a susceptibility to false positive identifications in deletion strains affected by plasmid segregation (e.g., *iml3*Δ, *chl1*Δ, or *ctf18*Δ) and *GCN4* activation (e.g., *tan1*Δ or *oca4*Δ), suggesting that this combination is highly sensitive to copy number variation and alteration in the transcription of the *ALR1* promoter (detailed in Discussion). Additionally, outside the core NMD factors, no overlap was observed between the secondary screens of pALR1_LEU2_PGA1 and pTDH3_HIS3_PGA1, with one exception; *tuf1*Δ, which was common in the secondary screen NMDv18-HISV2 and primary screen of NMD17-Leu2. However, this mutant dropped out during the secondary pooled screen using pTDH3_HIS3_PGA1. The identification of only core NMD genes aligns with a recent prepublication (Lobel and Ingolia 2024), which similarly identified core NMD genes along with a few essential genes that could not be screened using our current haploid deletion library. However, the inability of pALR1_LEU2_PGA1 to detect deletions with weaker effects on NMD highlights the limitations of this plasmid.

In contrast, the pTDH3_HIS3_PGA1 plasmid successfully captured deletions with weaker impacts on NMD, specifically *ebs1*Δ and *nmd4*Δ. Consistent with another recent NMD screen in *S. cerevisiae* (Pacheco et al. 2024), we identified *TMA20* and *EAF7* as potential factors influencing NMD. While we did not identify the *vps9*Δ mutant, as reported in the previous study, we instead found *vps21*Δ, which is involved within the same pathway. This makes *VPS21* along with *TMA20* interesting candidates for further characterization. The identified mutants classified by identification plasmid can be found in Supplemental Table S9.

### Characterization of candidate mutants

**FIGURE 4.**
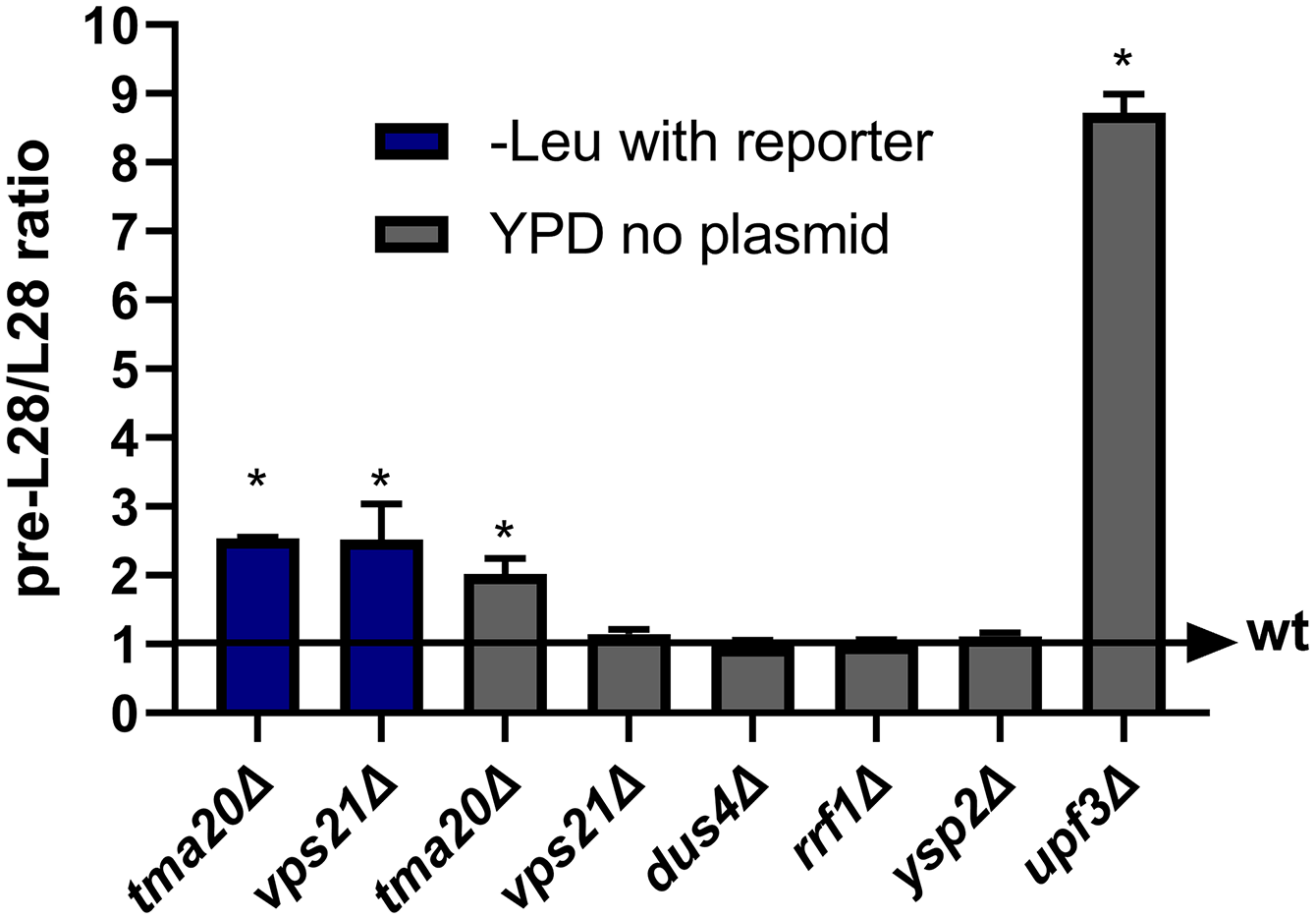
Verification of NMD defects in hits from finalized mutant screen. Quantitative PCR for the ratio of unspliced precursor of *RPL28* mRNA to the mature *RPL28* mRNA. *TAF10* was used for normalization. Error bars depict standard deviation (*n* = 3). Statistically significant results using a single sample *t*-test are marked with (*). Each tested strain is shown on the *x*-axis, and the column color indicates the condition. The wt baseline value of one is depicted as an arrow.

Next, we wanted to verify the candidates using an independent system. RT-qPCR for known NMD targets can be used in this case with RNA levels serving as a proxy for change in the half-life. A previous assay that utilizes the ratio of the unspliced precursor of *RPL28* mRNA, a known NMD target, to the mature form of *RPL28* mRNA (Dehecq et al. 2018) was used to assess whether the identified mutants were specific to the reporter or were truly influencing NMD. Mutants *tma20*Δ, which was reported to influence NMD while this work was ongoing (Pacheco et al. 2024), *ysp2*Δ, *rrf1*Δ, *dus4*Δ, and *vps21*Δ were selected for assessment since they had the clearest phenotypes. The mutant strains that did not carry a plasmid-borne reporter were tested in YPD media. We found that all mutants failed to meet our criteria for significance (fold change ratio of at least 2 and *P* < 0.05) with the exception of *tma20*Δ, which was borderline significant (Fig. 4). We considered that this could be an effect of the media. However, the previous report that identified *tma20*Δ (Pacheco et al. 2024) used complex media with galactose as the carbon source, indicating that minimal media is not a requirement for the mutants to test positive. This was further confirmed by repeating *RPL28* mRNA measurement in the *vps21*Δ and *tma20*Δ mutants in leucine drop-out media with the yeast carrying pRS314 LEU2 oriTP, a centromeric plasmid that does not harbor an NMD reporter (Moriguchi et al. 2013). The results (Supplemental Fig. S7) were similar to the results obtained with growth in YPD (Fig. 4) with *tma20*Δ testing significant and *vps21*Δ not, thus completely ruling out an effect of the media. Hence, we considered that this could be an effect of overexpression of an NMD-sensitive target since both in this and the previous work (Pacheco et al. 2024), the strong *TDH3* and *GAL* promoters were used to drive expression of the target. This indicates that the identified mutants could be affecting NMD only under conditions in which NMD is weakened by strong expression of an NMD-sensitive transcript. Mutants *tma20*Δ and *vps21*Δ were selected to test the hypothesis of the mutation affecting NMD only when it is weakened. Both strains carrying pTDH3_HIS3_PGA1 were grown in leucine drop-out media and tested again for effects on the unspliced precursor of *RPL28*. This time *vps21*Δ tested positive and *tma20*Δ had a higher fold change ratio (Fig. 4). This points to the possibility that the other mutants identified may behave in a similar manner.

### Mutations affecting ribosome recycling impair NMD

In mammalian cells, a CRISPR screen identified ribosome recycling factor ABCE1 as influencing NMD, as well as several factors involved in lysosome acidification (Zhu et al. 2020). It was further shown that the dependence on lysosome acidification was mediated through its effect on iron uptake, as supplementing iron restored NMD in cells with a defect in lysosome acidification. ABCE1 is an iron-sulfur (Fe–S) cluster protein, and in cells with defective lysosomal acidification, the ABCE1 levels were reduced. These observations implicated lysosome acidification and iron uptake in NMD through maintaining functional ABCE1 (Zhu et al. 2020). We hypothesized that analogously, having found the ribosome recycling factor Tma20 and the Rab protein Vps21 in our screen, a functional link could exist in yeast between NMD, the ABCE1 yeast ortholog Rli1, iron uptake, and vacuolar acidification. Vps21 is implicated in the transport of several components of the vacuolar ATPase that is responsible for vacuolar acidification (Gerrard et al. 2000).

**FIGURE 5.**
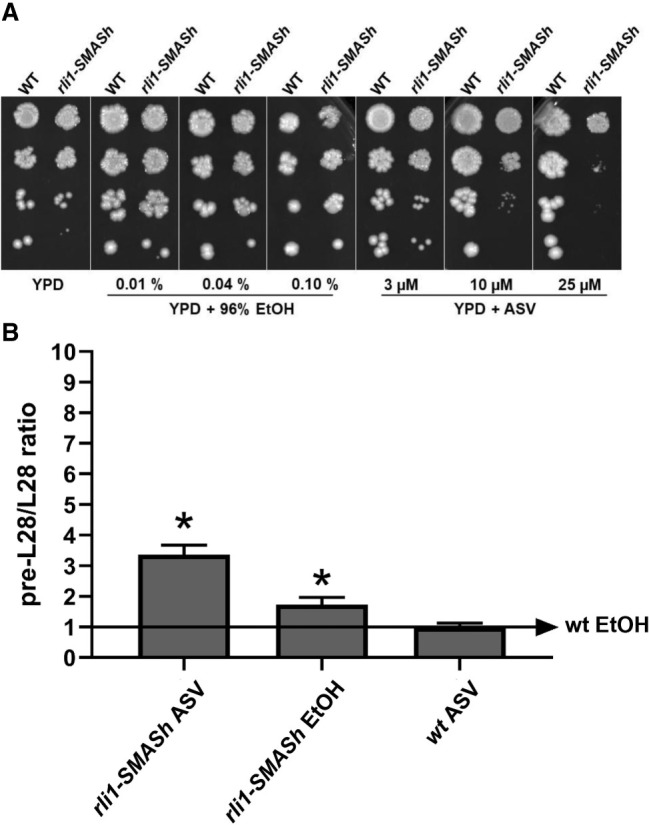
Verification of NMD defect in *rli1-*SMASh. (*A*) Growth inhibition of cells expressing *rli1-SMASh* in different ASV concentrations. Cells in logarithmic phase (OD_550 nm_ = 0.5) were spotted in serial 1:10 dilutions on YPD agar with the indicated additions. ASV was added to the indicated final concentrations. Control plates contain the ethanol solvent at the same concentrations as the plates with ASV at the corresponding concentrations. (*B*) qPCR of *RPL28* mRNA. Procedure and designations as in Figure 4. Each tested strain is shown on the *x*-axis. All samples are shown as relative level of the targeted mRNA species compared to the wt in medium plus 0.04% ethanol but without ASV. ASV was added to 10 µM where indicated. The baseline value of one, from wt in medium plus 0.04% ethanol, is depicted as an arrow. The 1.7-fold change for *rli1-SMASh* without ASV is marked with (*), since even though it does not meet the stricter criteria for being a true positive of a greater than twofold change, it is statistically significant.

We expected Rli1 to be required for ribosome recycling and involved in NMD like its mammalian ortholog ABCE1. *RLI1* is an essential gene, so we created a self-excising degron-dependent *rli1-*SMASh allele that can be regulated by asunaprevir (ASV) (Chung et al. 2015; see Materials and Methods) to be able to study its impact on NMD. As expected, the viability of *rli1*-SMASh cells decreased with increasing ASV concentrations (Fig. 5A). For the qPCR experiments to determine NMD efficiency, we chose 10 µM ASV, which gave a moderate growth inhibition. In agreement with expectations, NMD efficiency, again measured as the ratio unspliced precursor of *RPL28* mRNA/mature *RPL28* mRNA, increased 3.4-fold relative to wt with the *rli1-*SMASh knockdown allele after 5 h with 10 µM ASV, while ASV treatment of the wt caused no change in the ratio (Fig. 5B). We point out that the degron tagging had a constitutive effect on protein stability, as non-ASV treated *rli1*-SMASh displayed a ratio increase of about 1.7-fold relative to wt. This indicates that even a minor reduction of Rli1 in the absence of ASV caused an appreciable accumulation in the precursor of *RPL28*, although the increase was smaller than with ASV (Fig. 5B).

## DISCUSSION

We have designed a set of reporters that couple the cellular NMD status to growth. The utility of the system was demonstrated through identification in genome-wide screens of all previously known NMD components ranging from strong phenotypes (core NMD proteins) to weak phenotypes (*ebs1*Δ and *nmd4*Δ). We characterized two different reporters in the yeast deletion library and identified the potential weaknesses that are associated either with the level of background growth permitted by the given reporter or the promoter used. Use of the *ALR1*/*LEU2*-based reporter (pALR1_LEU2_PGA1) showed higher background growth relative to the *TDH3*/*HIS3*/*PGA1* (pTDH3_HIS3_PGA1) combination and lowered sensitivity since we could not detect a phenotype for neither *ebs1*Δ nor *nmd4*Δ using that combination. Moreover, pALR1_LEU2_PGA1 is more sensitive to mutations affecting plasmid copy number as demonstrated by the identification of various segregation mutants with this plasmid. Additionally, deletions that cause *GCN4* activation were identified as hits using pALR1_LEU2_PGA1, although it is very unlikely that they are associated with NMD (e.g., *tan1*Δ or *oca4*Δ) (De Zoysa and Phizicky 2020; Decourty et al. 2021). More probably, they increase transcription from the *ALR1* promoter, which is upregulated in response to *GCN4* activation (Hackett et al. 2020). An alternative explanation for the *tan1*Δ results could be related to the role of Tan1 in the formation of N^4^-acetylcytidine in tRNAs (Xu et al. 2018), with potential effects on translation. On the other hand, the pTDH3_HIS3_PGA1 reporter exhibited high sensitivity (all known NMD factors were identified) and very low background growth. Based on the above, it is not surprising that outside of the core NMD factors, there was no overlap in the hits identified by the different reporters. Therefore, hits selected for further validation came from the pTDH3_HIS3_PGA1 screen since it was able to identify weak NMD factors.

A small number of NMD “influencing” candidates were identified. We selected five candidates with a clear phenotype for testing via the detection of the changes in the unspliced precursor of *RPL28* mRNA. The majority of tested mutants did not yield a positive result when grown in complex media without the reporter, with the exception of *tma20*Δ. We reasoned that the media composition is unlikely to be the reason behind this. Rather, the phenotype of these mutants only becomes apparent when NMD is weakened with strong expression of an NMD target such as the one present in PTDH3_HIS3_PGA1 (carrying a long 3′ UTR from the *PGA1* gene). This hypothesis was tested using *tma20*Δ and *vps21*Δ. The results confirmed our line of thought since an increase in the unspliced precursor was seen in *vps21*Δ only when the plasmid was present, and this change became higher in *tma20*Δ as well when the plasmid was introduced. The results with these mutants indicate that a component that is usually not limiting for NMD becomes limiting and the growth phenotype manifest when NMD is weakened with the strong expression of an NMD substrate. This observation is an important consideration when further designing NMD reporters, as to the best of our knowledge this effect has not been reported elsewhere.

Vps21 belongs to the Rab family of small Ras-like GTPases and is homologous to mammalian Rab5 (Gerrard et al. 2000). This protein functions in the delivery of various proteins to the vacuole, including the Vma proteins, which are required for vacuolar acidification by the vacuolar ATPase complex (Anraku et al. 1992). Deletion of *VPS21* strongly reduces vacuolar acidification (Riechers et al. 2009) due to the mislocalization of the Vma proteins (Aufschnaiter and Buttner 2019). A CRISPR-based screen in mammalian cell lines found that acidification of lysosomes (the equivalent of the yeast vacuole) is linked to NMD through the disruption of iron homeostasis, which is turn reduces the level of the Fe–S cluster containing protein ABCE1 required for ribosome recycling (Zhu et al. 2020). We speculate that similar to the observation in mammalian cell lines, disruption of vacuolar acidification leads to a reduction in the activity of ribosome recycling factor Rli1 (the yeast Fe–S cluster-containing homolog of ABCE1), thus reducing NMD efficiency. It is of note that we did not directly identify any *vma* mutants as affecting NMD in the screen as was observed in mammalian cell lines; this is due to the absence of the majority of the *vma* deletion mutants from our constructed library, most likely due to their poor growth.

The *tma20*Δ mutation like *vps21*Δ is likely affecting NMD through ribosomal recycling (Pacheco et al. 2024). Tma20 (the yeast homolog of MCT-1) functions in the final steps of ribosomal recycling by dissociating the mRNA and tRNA from the 40S subunit to prevent translational reinitiation (Young et al. 2018). In either case (*vps21*Δ or *tma20*Δ), the question that needs to be asked is: How is ribosomal recycling linked to NMD?

Ribosomes that are not recycled by Rli1/ABCE1 can reinitiate in the 3′ UTR (Young et al. 2015). This reinitiation can abrogate NMD in mammalian cell lines through the clearing of the exon junction complex (EJC), which is required for NMD in the first round of translation; however, this does not explain how NMD is circumvented in subsequent rounds of translation or in yeast since they are not dependent on EJC for NMD activation. An alternative explanation comes from the observation that in mammalian cell lines UPF1 can bind along the long 3′ UTR prior to the initiation of decay and await activation (Hogg and Goff 2010). In this scenario, the unrecycled ribosomes displace UPF1 when moving through the UTR hence abrogating NMD. The latter scenario offers an explanation that is both applicable to any round of translation in mammalian cell lines and yeast since it is not reliant on the clearing of EJCs, although the binding of Upf1 to the 3′ UTR has not yet been demonstrated in yeast. It is important to note that Upf1 itself can recycle ribosomes at PTCs (Serdar et al. 2020). It was previously speculated that the ribosomal recycling could be dictated by two different pathways depending on whether the stop codon is in a normal context or premature, with each pathway being dependent on Rli1 and Upf1, respectively (Celik et al. 2015). Similar reasoning has been put forward regarding *S. cerevisiae* (Pacheco et al. 2024). We speculate that these pathways for ribosomal recycling might not be exclusive for one another and rather support each other to facilitate rapid clearing of NMD targets; however, additional work needs to be done to determine the exact mechanism. Here, we demonstrate a role for yeast *RLI1* in NMD. Its mechanistic function remains to be delineated in future studies.

Finally, it is important to discuss the strengths and weaknesses of our system relative to the fluorescent reporter system described by Pacheco et al. (2024), published while this work was ongoing. We designed our system to be plasmid-borne to simplify the handling although that introduces issues relating to plasmid copy number instability affecting the false positive identification rate, and this issue was more prominent with the low expression plasmid (pALR1_LEU2_PGA1). However, this problem can be easily resolved by excluding genes that affect plasmid segregation from the identified mutant lists should they test positive; alternatively, this problem can be resolved altogether by integrating the construct. On the positive side, our system is much simpler than the fluorescent reporter system (Pacheco et al. 2024) as no specialized equipment is needed to use our growth-based system. Depending on the particular focus of a study, it is possible to perform a single genome-wide screen for NMD factors, using just one of the reporters carrying an NMD trigger of the structure of interest. In either case, the choice of which system to use will be dependent on a given laboratory's capability and experimental design.

Overall, we describe a simple to use system for monitoring the cellular NMD status and provide direct evidence for the involvement of *RLI1* in NMD in yeast. Application of the system in the deletion collection revealed the possibility that vacuolar acidification might influence NMD under conditions in which NMD is weakened, and added support to the recent observation that the *tma20*Δ deletion alters NMD efficiency. In the future, we hope to utilize our system for assessing NMD under various environmental conditions. We envision that the combination of the efficient methodology described here and other global methods, such as SLAM-seq, will help clarify the workings of NMD in finer details.

## MATERIALS AND METHODS

### Strains and media

YPD (2% glucose, 2% peptone, and 1% yeast extract) was used for routine culturing of strains not carrying plasmids and selection for antibiotic resistance during strain construction. Synthetic drop-out medium (SD) was used for plasmid selection and consisted of 0.19% yeast nitrogen base, 0.5% ammonium sulfate, 2% glucose, and 0.077% Complete Supplement Mixture with appropriate amino acid/s omitted (ForMedium). G418 and hygromycin were used at a concentration of 200 µg/mL when required. Agar was used at a concentration of 2% when using solid media. Synthetic drop-out media supplemented with 2% galactose and 2% raffinose and lacking the amino acid required for the plasmid selection and glucose (SGR) was used during library construction. Growth of cultures was carried out at 30°C and liquid cultures were grown in a rotary shaker at 200 rpm.

### Constructions of strains and plasmids

Phusion Hot Start II (Thermo Fisher) polymerase was used for amplifying all the products used for either plasmid or strain construction. Either restriction ligation or NEBuilder HiFi DNA Assembly Master Mix was used for the construction of the plasmids. Deletion strains were created by transforming PCR products with 50 bp of homology on both sides of the region specific for each deletion into the appropriate strain using lithium acetate/PEG/ssDNA transformation (Gietz and Woods 2002).

Details for the construction of each plasmid and strain along with the sequences of primers and synthetic fragments used can be found in Supplemental Methods S1.

### CPSase growth test

Overnight cultures of test strains carrying plasmid pHA_URA2ΔCPSaseV3 were washed with water and diluted to OD_600 nm_ = 0.1 into 100-well honeycomb plates (Labsystems Oy) in a final volume of 200 µL using two different media. The first medium was the control condition, while the second media was *CPA1* repression medium. The CPSase control medium consisted of 0.19% yeast nitrogen base, 0.5% ammonium sulfate, 2% glucose, and for the *CPA1* suppression condition, the ammonium sulfate was replaced with arginine at 1 mg/mL. Strains were cultivated for 3 days with the low shaking setting at 30°C with 20-min measurement intervals using a wide band filter (420–580 nm).

### Growth curves of NMD reporters

Overnight cultures were made in SD with appropriate dropout of supplements. Before the start of the experiment, the medium was removed and the pellet resuspended in 1 mL SD with appropriate dropout for the NMD reporter auxotrophies. Subsequently, the cultures were diluted to OD_600 nm_ = 0.1 into 100-well honeycomb plates (Labsystems Oy) in a final volume of 200 µL using the same media used for the resuspension. Strains were cultivated for 3 days with the low shaking setting at 30°C with 20-min measurement intervals using a wide band filter (420–580 nm).

### Library construction and screening

NMD reporters were introduced to yeast deletion library mutants using the method developed by Reid et al. (2011) using the HDA RoToR robot (Singer Ltd.) with minor modifications. W8164-2B (Reid et al. 2011) cells carrying the NMD reporters were previously cultivated on SD -LEU or SD -HIS depending on the reporter and mated to the deletion mutants overnight in 384 arrays by overlaying the two strains on YPD plates. Subsequently, the mated array was replica plated to SGR media for two sequential rounds. Following the second purification on the SGR media, the array was replica plated to SGR media supplemented with 50 µg/mL uracil and 1 mg/mL 5-FOA. Strains that grew after 5-FOA counter selection were transferred to a 384-well plate containing 50 µL of SD -HIS supplemented with 15% glycerol and frozen at −80°C until the screening started for the first library. The second library was kept on the last selection plate at 4°C until the start of the screen.

The 384-well plates containing the constructed first library were thawed at room temperature. Subsequently, two precultures from each library plate were made on SD plates using 1536 density. Each preculture contained three replicates of each mutant interleaved with a wt control at every fourth position, giving a total of six replicates per mutant. After growing the cultures for 4 days, they were replica plated using 1536 pads on SD plates that only select for the plasmid and on SD -HIS -LEU plates to screen for the NMD reporter's phenotype. The plates carrying the freshly pinned cultures were scanned using Scan-o-matic framework version 2.2 (Zackrisson et al. 2016) every 20 min for 4 days at 30°C and the pixel intensities were used for the constructions of growth curves. Subsequently, a relative growth rate (relative to the wt control at every fourth position) was extracted for each position using the same software. The physical setup and quality checks were done as described previously (Alalam et al. 2020) with the exception of using calibrated pixel intensities transformed into population sizes using the formula for *S. cerevisiae* detailed previously (Zackrisson et al. 2016). The second screen was carried out by pinning the mutant arrays into quadruplets on SD -HIS -LEU plates and carrying out the screen as above but at 6 h scanning intervals.

### Screen analysis

For the first library, an interaction score was calculated for each mutant in the screening plate by subtracting the normalized growth of the screening plate from the control plate. Each mutant position was paired to its cognate position in the control plate resulting in six interactions score per mutant. In Scan-o-matic, the normalized growth rates are negative when the position being compared had better growth than the control strain at the fourth position (meaning that this position has a reduced generation time relative to the control and hence a negative score), while a positive normalized growth rate means the opposite. The subtraction described above will lead to a positive score if the mutant grew better on the test plate and a negative score if the mutant grew worse. Mutants that had at least three out of six measurable interaction scores were further tested using a single sample *t*-test versus the mean of the control population and the *P*-values adjusted using FDR. All tests and data summaries were made using R 4.2, while growth curve graphs were made using GraphPad Prism 9. For the second library, the images at 48 h were visually inspected and any quadruplets that displayed any growth were selected. Similarly, the candidates from the final screen were selected by visual inspection at 48 h but with an increase number of replicates (*n* = 16).

### Reverse transcription and quantitative PCR

RNA was extracted from log phase cells using Quick-RNA Fungal/Bacterial Miniprep Kit (Zymo research) and treated with TURBO DNase (Thermo Fisher). Five hundred nanograms of RNA was subjected to reverse transcription using iScript Reverse Transcription Supermix (Bio-Rad). The resulting cDNA was diluted 10-fold and prepared for amplification using iQ SYBR Green Supermix (Bio-Rad). The amplification was done using CFX connect thermal cycler (Bio-Rad) using the following programs: 95°C for 4 min followed by 40 cycles of 95°C for 10 sec and 55°C for 45 sec. The primers used for the reference gene (*TAF10*) are described in Teste et al. (2009), and the primers used the spliced and unspliced form of *RPL28* are described in Dehecq et al. (2018). Single sample *t*-test vs. the baseline value was used to determine statistical significance.

## SUPPLEMENTAL MATERIAL

Supplemental material is available for this article.
